# Late Cardiac Pathology in Severe Covid-19. A Postmortem Series of 30 Patients

**DOI:** 10.3389/fcvm.2021.748396

**Published:** 2021-10-15

**Authors:** Ana Ferrer-Gómez, Héctor Pian-Arias, Irene Carretero-Barrio, Antonia Navarro-Cantero, David Pestaña, Raúl de Pablo, José Luis Zamorano, Juan Carlos Galán, Belén Pérez-Mies, Ignacio Ruz-Caracuel, José Palacios

**Affiliations:** ^1^Pathology Department, University Hospital Ramón y Cajal, Madrid, Spain; ^2^Faculty of Medicine, Alcalá University, Alcalá de Henares, Spain; ^3^Anaesthesiology and Surgical Critical Care Department, Hospital Universitario Ramón y Cajal, Madrid, Spain; ^4^Instituto Ramón y Cajal for Health Research (IRYCIS), Madrid, Spain; ^5^Medical Intensive Care Unit, Hospital Universitario Ramón y Cajal, Madrid, Spain; ^6^Cardiology Department, Hospital Universitario Ramón y Cajal, Madrid, Spain; ^7^Centro de Investigación Biomédica en Red de Enfermedades Cardiovasculares (CIBERCV), Instituto de Salud Carlos III, Madrid, Spain; ^8^Microbiology Department, Hospital Universitario Ramón y Cajal, Madrid, Spain; ^9^Centro de Investigación Biomédica en Red en Epidemiología y Salud Pública (CIBERESP), Madrid, Spain; ^10^Centro de Investigación Biomédica en Red de Cáncer (CIBERONC), Instituto de Salud Carlos III, Madrid, Spain

**Keywords:** COVID-19, SARS-CoV-2, heart, cardiac pathology, autopsy

## Abstract

The role of SARS-CoV-2 as a direct cause in the cardiac lesions in patients with severe COVID-19 remains to be established. Our objective is to report the pathological findings in cardiac samples of 30 patients who died after a prolonged hospital stay due to Sars-Cov-2 infection. We performed macroscopic, histological and immunohistochemical analysis of the hearts of 30 patients; and detected Sars-Cov-2 RNA by RT-PCR in the cardiac tissue samples. The median age of our cohort was 69.5 years and 76.6% were male. The median time between symptoms onset and death was 36.5 days. The main comorbidities were arterial hypertension (13 patients, 43.3%), dyslipidemia (11 patients, 36.7%), cardiovascular conditions (8 patients, 26.7%), and obesity (8 patients, 26.7%). Cardiovascular conditions included ischemic cardiopathy in 4 patients (13.3%), hypertrophic cardiomyopathy in 2 patients (6.7%) and valve replacement and chronic heart failure in one patient each (3.3%). At autopsy, the most frequent histopathological findings were coronary artery atherosclerosis (8 patients, 26.7%), left ventricular hypertrophy (4 patients, 13.3%), chronic epicardial inflammation (3 patients, 10%) and adipose metaplasia (2 patients, 6.7%). Two patients showed focal myocarditis, one due to invasive aspergillosis. One additional patient showed senile amyloidosis. Sars-Cov-2 RNA was detected in the heart of only one out of 30 patients, who had the shortest disease evolution of the series (9 days). However, no relevant cardiac histological alterations were identified. In present series, cardiac pathology was only modest in most patients with severe COVID-19. At present, the contribution of a direct effect of SARS-CoV-2 on cardiac lesions remains to be established.

## Introduction

Coronavirus-19 disease (COVID-19), caused by the new coronavirus SARS-CoV-2 has become a global health challenge in our time (1). It is known that SARS-CoV-2 affects mainly the respiratory system, with a spectrum of clinical manifestations ranging from asymptomatic to mild illness with fever and fatigue (80% of symptomatic patients) (2). However, in the most severe cases, which represent around 5%, it can lead to respiratory distress syndrome that requires ventilatory support (3, 4). As cardiovascular complications including myocarditis, acute myocardial infarction, and exacerbation of heart failure are present in patients suffering from other respiratory viral infections, such as influenza virus each annual epidemic period, special attention has been paid to a possible heart involvement by SARS-CoV-2 since the beginning of the pandemic (5). In fact, reported clinical cardiac manifestation in COVID-19 patients included right heart dilatation and dysfunction, myocarditis, cardiac fibrosis, arrhythmias, endothelial dysfunction, dysautonomia, and thrombotic events (6). Moreover, it has been suggested that patients with a history of cardiovascular disease or cardiovascular risk factors such as hypertension, dyslipidemia or obesity are strongly associated to severe symptoms and higher mortality rate in patients infected by SARS-CoV-2 (2, 7). The possible pathophysiological mechanisms by which SARS-CoV-2 would cause damage to the myocardium and vascular endothelium include a direct myocardial injury due to viral invasion, a damage secondary to hypoxemia as consequence of respiratory failure, infarct secondary to thrombosis, as well as a dysregulated immunological response (known as cytokine storm) (8).

Since autopsies are the gold standard procedure to settle the underlying pathophysiology of the diseases (9), several autopsy series of patients who died from COVID-19 have been reported in the last months. In addition, some studies have reviewed the cardiac lesions reported in those series (10–12). However, our information regarding cardiac pathology is limited to about 700 hearts and, despite these studies, there is still limited and controversial data about the histopathological cardiac findings in patients with SARS-CoV-2 infection. Previous series are mostly formed by patients who died during the acute illness. Thus, the longest duration of illness in the cohorts reviewed by Roshdy et al. (10) was 52 days with a median duration from symptoms onset to death of 12 days (range, 0–52 days, *n* = 98), which means that the long-term evolution or complications of the disease were not covered by this review.

The objective of our study is to present the histopathological cardiac lesions in a series of 30 autopsies performed in patients who died by severe COVID-19 with relative long-term evolution from symptoms onset (median 36.5 days, range 9–108 days).

## Materials and Methods

### Autopsy Procedure and Clinical Data

This is a retrospective analysis of the macroscopic and histological findings in the hearts of all autopsies performed on patients with COVID-19 in University Hospital Ramón y Cajal (Madrid, Spain), from April 2020 to April 2021 (*n* = 30), representing ~3% of patients who died from COVID-19 during this period. The Research Ethics Committee approved the study (reference: Necropsias_Covid19; 355_20). All the deceased patients were diagnosed of SARS-CoV-2 infection confirmed by RT-PCR nasopharyngeal swab test. Demographic and clinical data were collected from the electronic medical records.

These consecutive autopsies were requested by the medical staff according to clinical interest. Most autopsies (*n* = 25, 83%) corresponded to patients with severe respiratory diseases and were requested by ICU staffs. Consequently, the series does not represent the complete spectrum of causes of death attributable to COVID-19 (Table 1). All autopsies were consented by patients' relatives and carried out according to safety protocols, in a negative pressure autopsy room, using personal protection equipment, as previously reported (13).

**Table 1:** Demographic, clinical, and laboratory findings.

**Demographics**	**Total**		**30 (100%)**
Male, *n* (%)			23 (76.67%)
Age	Median (IQR)		69.5 (12.25)
	Min, Max		52, 91
Weight	Median (IQR)		76.5 (14)
	Min, Max		53, 109
Days from initial symptoms	Median (IQR)		36.5 (17.5)
	Min, Max		9, 108
Hospitalization days	Median (IQR)		29.5 (18)
	Min, Max		3, 102
Patients admitted to ICU, *n* (%)			26 (86.67%)
ICU days	Median (IQR)		21 (15)
	Min, Max		12, 95
Mechanical ventilation, *n* (%)			26 (86.67%)
**Comorbidities**			
Hypertension, *n* (%)			13 (43.33%)
Dyslipidemia, *n* (%)			11 (36.67%)
Cardiovascular condition, *n* (%)			8 (26.67%)
•Ischemic cardiopathy			4 (50.00%, *n =* 8)
•Hypertrophic cardiomyopathy			2 (18.18%, *n =* 8)
•Valve replacement			1 (12.50%, *n =* 8)
•Heart failure			1 (12.50%, *n =* 8)
Obesity, *n* (%)			8 (26.67%)
Malignancy, *n* (%)			6 (20.00%)
Chronic obstructive pulmonary disease/SAHS, *n* (%)			5 (16.67%)
Neurologic condition, *n* (%)			3 (10.00%)
Diabetes mellitus, *n* (%)			2 (6.67%)
Hepatic condition, *n* (%)			2 (6.67%)
Immunosuppression, *n* (%)			1 (3.33%)
**Clinical symptoms**			
Fever at admission, *n* (%)			24 (80.00%)
Dyspnea at admission, *n* (%)			22 (73.33%)
Cough at admission, *n* (%)			17 (56.67%)
Asthenia at admission, *n* (%)			13 (43.33%)
Diarrhea at admission, *n* (%)			4 (13.33%)
Anosmia/Ageusia at admission, *n* (%)			4 (13.33%)
Nausea/Vomiting at admission, *n* (%)			3 (10.00%)
Temperature in Celsius at admission	Median (IQR)		36.5 (0.6)
	Min, Max		35.5, 38
Oxygen saturation at admission	Median (IQR)		88 (14.3)
	Min, Max		70, 99
Heart rate at admission	Median (IQR)		100 (25)
	Min, Max		70–141
Arrythmia, *n* (%)	Previously diagnosed		2 (6.67%)
	During hospitalization		6 (20%)
**Department that requested the autopsy**			
Anesthesiology department, *n* (%)			18 (60%)
Medical intensive care unit, *n* (%)			7 (23.33%)
Internal medicine department, *n* (%)			2 (6.67%)
Geriatrics department, *n* (%)			2 (6.67%)
Pneumology department, *n* (%)			1 (3.33%)
**Cause of death (according to autopsy report)**			
Hypoxemia, *n* (%)			24 (80%)
Pancreatitis, *n* (%)			2 (6.67%)
Intestinal necrosis, *n* (%)			2 (6.67%)
Subarachnoid hemorrhage, *n* (%)			1 (3.33%)
Invasive aspergillosis, *n* (%)			1 (3.33%)
**Laboratory test at admission (normal values)**			
White cell count /μL (4–11 × 10^∧^3)	Median (IQR)		11.1 (9.90)
	Min, Max		0.01, 21.9
% Neutrophils (45–75)	Median (IQR)		85.75 (20.28)
	Min, Max		18.2, 96.9
Lymphocytes/μL (1–4.5 × 10^∧^3)	Median (IQR)		0.89 (0.47)
	Min, Max		0, 1.45
Creatinine mg/dL (0.3–1.3)	Median (IQR)		0.92 (0.37)
	Min, Max		0.48, 2.82
CRP mg/L (0–5)	Median (IQR)		188.20 (160.75)
	Min, Max		0, 0.8
Ferritin ng/mL (20–300)	Median (IQR)		1454.01 (1496.12)
	Min, Max		59.15, 5269.9
Lactate dehydrogenase U/L (140–240)	Median (IQR)		442 (274.25)
	Min, Max		333, 987
Platelets/μL (140–400 × 10^∧^3)	Median (IQR)		195 (146.25)
	Min, Max		22.3, 599
Fibrinogen mg/dl (150–400)	Median (IQR)		740 (31.65)
	Min, Max		295.1,740
APTT% (76–128)	Median (IQR)		84.85 (21.25)
	Min, Max		16.1, 131.3
PT s (9.7–12, 6)	Median (IQR)		12.20 (1.18)
	Min, Max		10.2, 49.2
D-dimer ng/mL (0–500)	Median (IQR)		1,164 (2,098)
	Min, Max		152, 14,493.41
Troponin at admission ng/ml (0–0.1)	Median (IQR)		0.00 (0.00)
	Min, Max		0, 0.8
Highest troponin during hospitalization ng/ml (0–0.1)	Median (IQR)		0.00 (0.00)
	Min, Max		0, 10.4
Natriuretic peptide pg/mL (<100)	Median (IQR)		81.80 (125.30)
	Min, Max		0.4, 2,288
IL6 pg/mL (0)	Median (IQR)		62.15 (121.04)
	Min, Max		1.27, 589
IL10 pg/mL (0)	Median (IQR)		7.94 (6.79)
	Min, Max		0.5, 54.44
IL12 pg/mL (0)	Median (IQR)		1.19 (2.40)
	Min, Max		0, 6.66
**Pathologic findings**	**Partial heart examination (*****n** **=*** **14)**	**Complete heart examination (*****n** **=*** **16)**	**Total (*****n** **=*** **30)**
**Heart**			
Coronary artery atherosclerosis, *n* (%)	2 (14.29%)	6 (37.5%)	8 (26.67%)
Left ventricle hypertrophy, *n* (%)	3 (21.43%)	1 (6.25%)	4 (13.33%)
Chronic epicardial inflammation, *n* (%)	0	3 (18.75%)	3 (10%)
Myocarditis, *n* (%)	0	1 (6.25%)	1 (3.3%)
Aspergillus myocarditis, *n* (%)	1 (7.14%)	0	1 (3.3%)
Senile amyloidosis, *n* (%)	1 (7.14%)	0	1 (3.3%)
Without significant alterations, *n* (%)	5 (35.71%)	8 (50%)	13 (43.33%)
**Lung**			
Patients with predominant pattern, *n* (%)	Normal lung		1 (3.3%)
	Exudative DAD		6 (20%)
	Proliferative/Organizing DAD		19 (63.3%)
	Fibrotic DAD		4 (13.3%)
Acute bronchopneumonia, *n* (%)			12 (40%)
Vascular thrombi, *n* (%)			20 (67%)
Endotheliitis, *n* (%)			13 (43%)

*Diffuse alveolar damage (DAD)*.

In the first 14 consecutive decedents, we took *in-corpore* representative sections from the heart, lungs, liver, kidney, pancreas, and bone marrow. In the rest of the patients, due to improved technical training, we extracted the complete heart and lung block, left kidney, spleen, and sections from the liver, pancreas and bone marrow. We extracted the brain in 10 patients.

After each procedure, the hearts were fixed in formalin for 24–48 h.

### Histopathology and Immunohistochemistry

**Figure 1:**
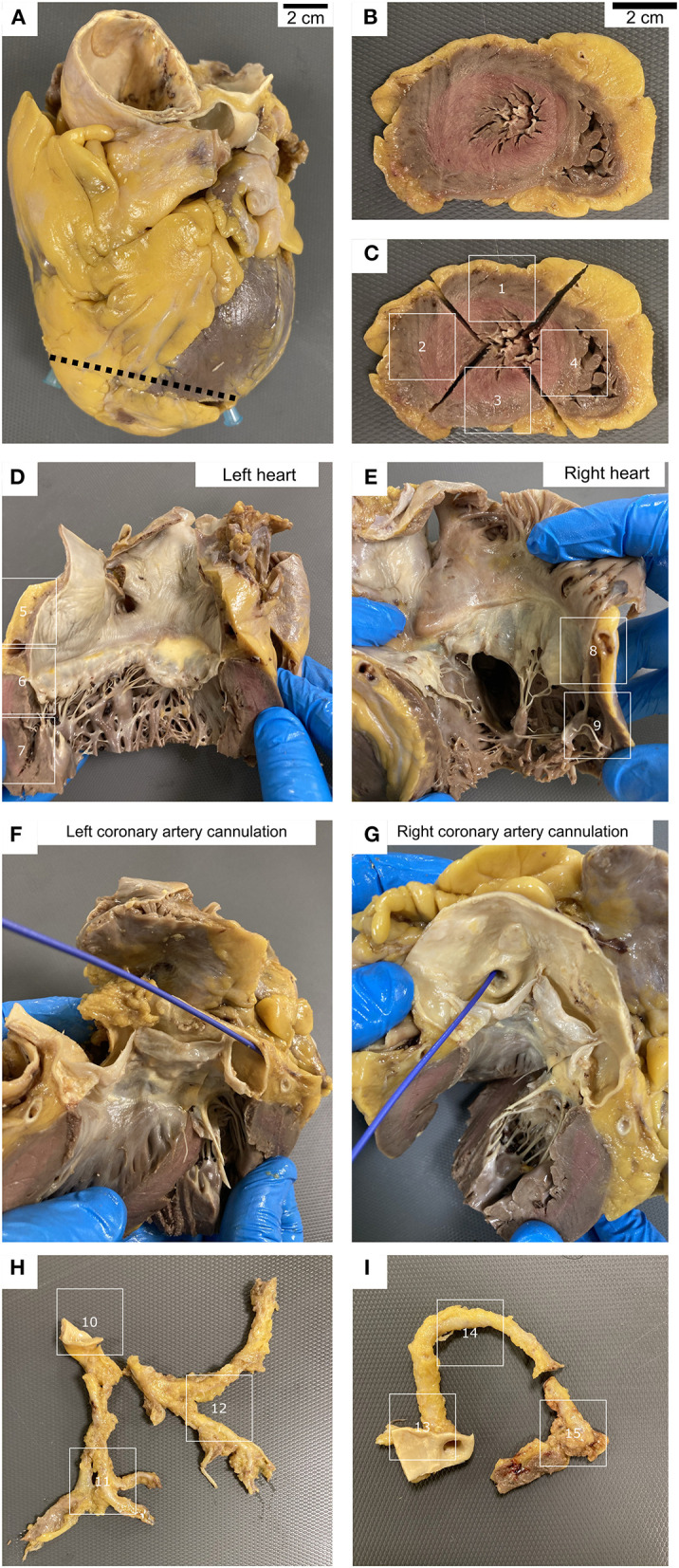
Heart grossing protocol. **(A–C)** Apex sections. **(D)** Left atria and ventricle sections, including mitral valve. **(E)** Right atria and ventricle sections, including tricuspid valve. **(F–I)** Dissection of the coronary arteries. Cannulation of left **(F)** and right **(G)** coronary arteries. Left **(H)** and right **(I)** coronary arteries sections.

For histological study, in the 14 first autopsies, we only took 5 representative sections corresponding to the anterior, lateral and posterior left ventricle walls, the septum, and right ventricle wall. In the rest of the patients, the complete heart and great proximal vessels were studied following the protocol depicted in Figure 1.

Hematoxylin and eosin stain was performed in all sections. In addition, when inflammatory infiltrates were present, special histochemical stains such as PAS, Grocott or Gram were done to rule out microorganisms such as bacteria or fungi. Congo red stain was performed in suspected cases of amyloid deposit.

To analyze the immune infiltrates, we performed immunohistochemistry for CD20, CD3, CD8, CD4, and CD68 (Agilent, Santa Clara, CA, USA) in sections with inflammation. Transthyretin immunohistochemistry (Agilent, Santa Clara, CA, USA) was also done to confirm senile amyloidosis.

### Sars-Cov-2 RNA Analysis

To investigate the presence of Sars-Cov-2 RNA, we took swabs samples from the left ventricle in 28 patients. In addition, we tested Sars-Cov-2 RNA in paraffin blocks from the left ventricle in all 30 patients, selecting those blocks with inflammatory infiltrates.

All swab samples were sent on the same day to the Microbiology Department for the detection of genomic SARS-CoV-2 RNA (gRNA). RNA extraction and reverse-transcription-polymerase-chain-reaction (RT-PCR) amplification were performed within 3 h after reception in the laboratory. RNA extraction was performed using MagmaxTM Core Nucleic Acid Purification Kit (Thermo Fisher, Waltham, United States) and gRNA SARS-CoV-2 was detected using TaqmanTM 2019 nCoV assay (Thermo Fisher, Waltham, United States).

For formalin-fixed-paraffin embedded (FFPE) samples, RNA was extracted from 10 sections of 5 μm obtained from paraffin blocks using RecoverAll Total Nucleic Acid Isolation Kit (Invitrogen), following the manufacturer's instructions. RNA quantity was measured fluorometrically with Qubit RNA high-sensitivity assay kit (Invitrogen, Waltham, MAS, USA).

### Literature Review

We have performed a non-systematic PUBMED review of autopsy series published in English including 4 or more patients not previously reported in the reviews by Halushka and Vander Heide (11), Roshdy et al. (10) and Kawakami et al. (12).

## Results

### Demographics

The main demographic, clinical and laboratory findings of the 30 patients are listed in Table 1. The median age of our cohort was 69.5 years (range 52–91). Twenty-three patients (76.6%) were male. The median time between admission and death was 29.5 days (range 3–102). The main comorbidities were arterial hypertension in 13 patients (43.3%), dyslipidemia in 11 patients (36.7%), cardiovascular conditions in 8 patients (26.7%), obesity in 8 patients (26.7%), and diabetes in 2 patients (6.67%). None of them were vaccinated.

Cardiovascular conditions included ischemic cardiopathy in 4 patients (13.3%), hypertrophic cardiomyopathy in 2 patients (6.7%) and mitral and aortic valve replacement and chronic heart failure in one patient each (3.3%). Two patients had a previous diagnosis of auricular flutter and auricular fibrillation, respectively. Six patients developed arrythmias during hospitalization, including supraventricular extrasystoles (6.7%), auricular flutter (3.3%), bundle branch block (3.3%), streaks of supraventricular tachycardia (3.3%), and self-limited periods of arrhythmias (3.3%).

### Pathology

The main pathological findings of this series are presented in Table 1.

#### Cardiac Pathology

##### Macroscopic Findings

Mean post-fixation weight of the heart, in 16 (53.3%) patients in whom the complete organ was studied, was 474.2 g (range 310–720) (normal weight 365 ± 71 g). Regarding macroscopic findings, serous pericardial effusion was evidenced in 8 patients (26.6%), left ventricular hypertrophy (>1.5 cm in diameter) was present in 4 patients (13.3%), adipose myocardial replacement in 2 patients (6.6 %), and the presence of a macrothrombus in left atrium in one patient (3.3%). Two hearts showed post-surgical changes; one showed mitral and aortic valve replacement and another a coronary artery bypass grafting. In 14 patients (47%) no macroscopically relevant alterations were identified in the heart.

##### Histopathology

**Figure 2:**
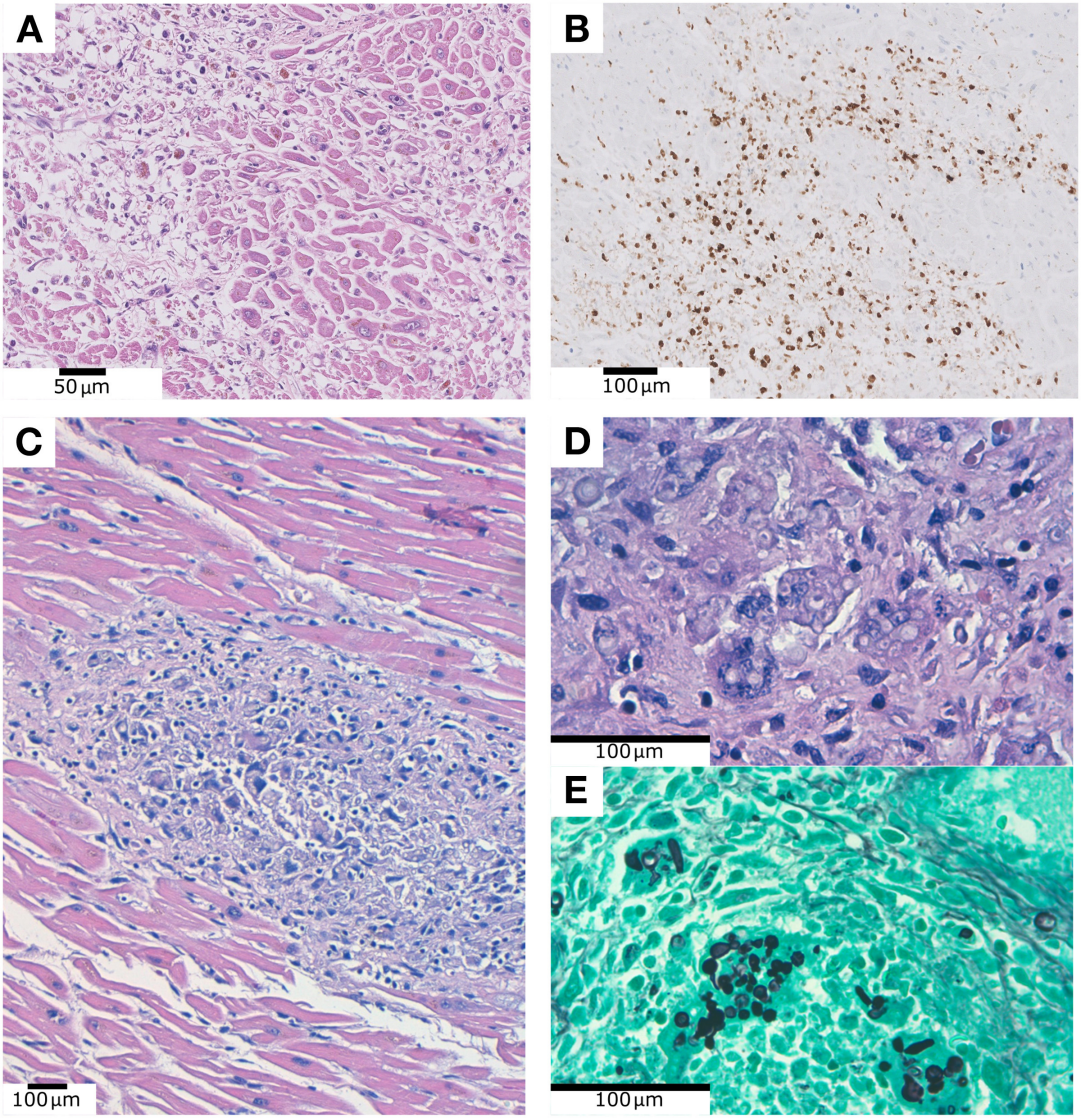
**(A)** Myofibrils necrosis associated with abundant inflammatory infiltration. **(B)** Same case as **(A)**, showing CD68 positive macrophages infiltrating the myocardium. **(C)** Fungal structures within the myocardium, associated to myofibrils necrosis and inflammation. **(D)** Same case as **(C)**, PAS technique. **(E)** Same case as **(C)**, Grocott technique.

The most frequent histopathological finding was coronary artery atherosclerosis (8 patients, 26.7%). Three patients (10%) showed focal chronic epicardial inflammation, that consisted of a slight lymphocytic mononuclear inflammatory infiltrate associated with reactive mesothelium. This infiltrate was mainly composed of T lymphocytes (CD3+) with a predominant cytotoxic phenotype (CD8+) that exceed the CD3+/CD4+ population. One patient (3.3%) revealed myofibrils necrosis associated with abundant macrophage infiltration and occasional CD3+ lymphocytes in an area of ~1 cm^2^ in the left ventricle, consistent with the diagnosis of myocarditis (Figures 2A,B).

In a 55-year-old male patient, without any comorbidity and 37 days of hospitalization, we observed the presence of ring-shaped fungal structures within the myocardium, associated to myofibrils necrosis and inflammation. This patient received corticosteroids and antibiotics, but no antifungal treatment. The fungal structures were positive for PAS and Grocott techniques (Figures 2C–E). They were also identified in lung and kidney parenchyma, where they were identified as *Aspergillus fumigatus* by postmortem microbiological culture and PCR. We did not observe intranuclear or intracytoplasmic inclusions in myocardial cells in none of our patients.

**Figure 3:**
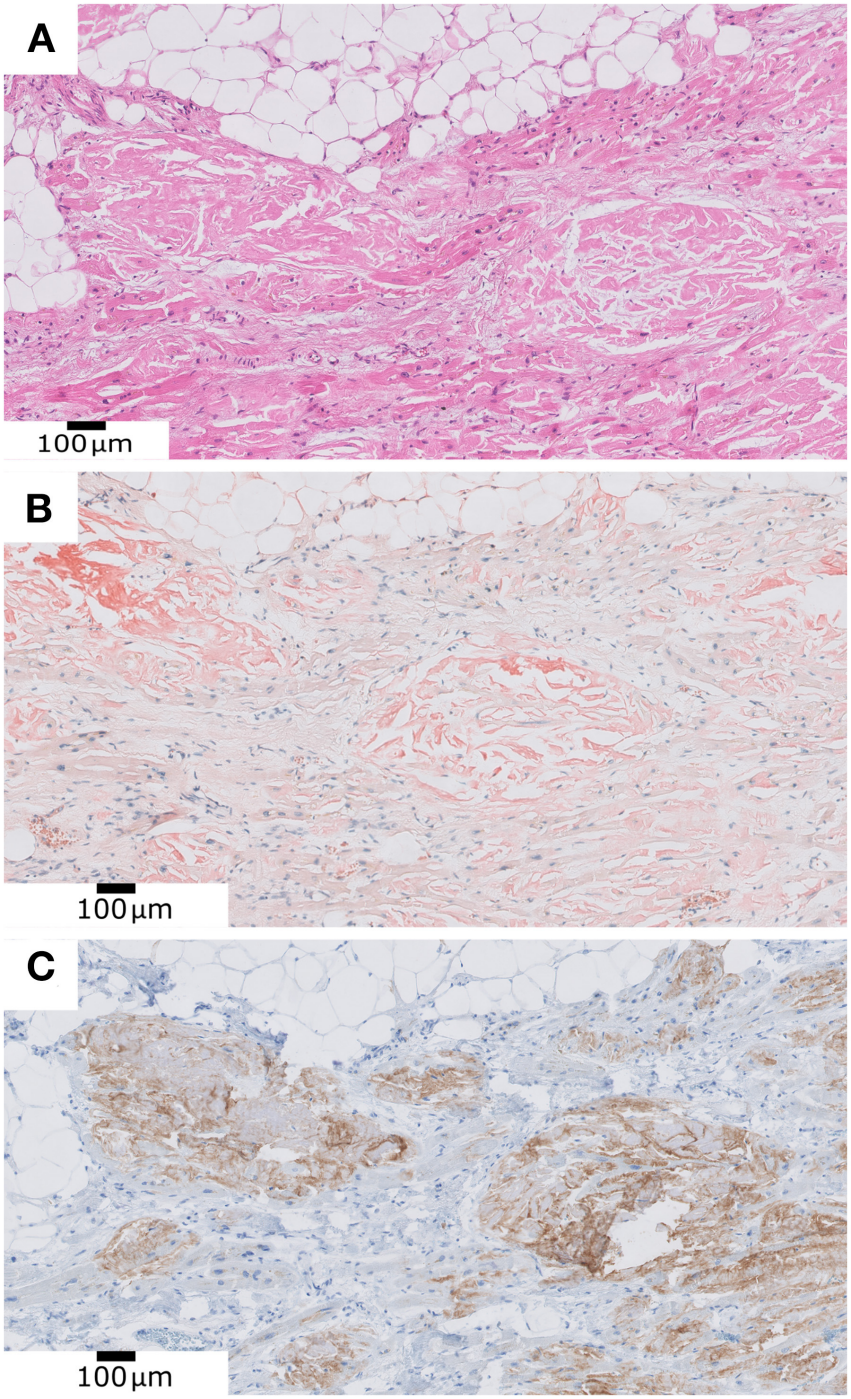
**(A)** Deposit of eosinophilic and amorphous material compatible with amyloid within the myocardium. **(B)** Positive Congo Red histochemical stain. **(C)** Positive transthyretin immunohistochemistry.

In a 90-year-old patient, suffering chronic cardiac failure, we evidenced a deposit of eosinophilic and amorphous material compatible with amyloid within the myocardium (Figure 3). This was confirmed with Congo red histochemical stain. This deposit was also identified in the cardiac and pulmonary vessels, pericardial adipose tissue, kidney and bone marrow. After performing immunohistochemical staining, the amyloid material was positive for transthyretin and negative for Kappa, Lambda and Amyloid AA, rendering the diagnosis of senile amyloidosis.

No histologically relevant alterations were found in 13 patients (43.3%), according to their age and clinical status.

##### Sars-Cov-2 RNA Analysis

All myocardial swabs except one [Cycle threshold (Ct) = 28] were negative for Sars-Cov2. The only positive case was also positive in the study of the FFPE tissue (Ct = 33), and Ct = 20 in nasopharyngeal sample obtained in the same autopsy. However, this patient did not show any relevant macroscopic or histopathological cardiac lesion. Interestingly, in spite of the absence of Sars-Cov-2 RNA in cardiac samples, all patients showed at least a positive result from the nasopharynx or lung in autopsy samples (manuscript in preparation).

##### Literature Review

Supplementary Table 1 compares the clinical and pathological findings reported by Halushka and Vander Heide (11) in their review of 293 cases and our review of 280 additional cases, including the 30 patients here reported.

## Discussion

In this study, we report the cardiovascular findings in the autopsies of 30 patients with severe COVID-19. Our results indicate a modest involvement of the heart in these patients, being the most frequent histopathological findings coronary artery atherosclerosis (8 patients, 26.7%), left ventricle hypertrophy (4 patients, 13.3%), chronic epicardial inflammation (3 patients, 10%), focal myocarditis (1 patient, 3.3%), and myocarditis due to Aspergillus (1 patient, 3.3%).

To the best of our knowledge, this is the autopsy series with the longest disease duration in which the heart has been histopathologically analyzed, and our results are in accordance with other studies with a shorter time of disease evolution.

Regarding duration of disease, in the review by Roshdy et al. (10), the median duration of prehospital symptoms (*n* = 82) and hospital stay (*n* = 158) were 5 (IQR, 2–7) and 6 days (IQR, 3–10), respectively. In total, the median duration from the onset of symptoms to death was 12 days (range, 0–52 days, *n* = 98). In the review by Halushka and Vander Heide (11), the median number of days from diagnosis to fatality was 10 (range 1–51 days). In contrast, our series included patients with a median disease duration from symptoms onset to death of 36.5 and 29.5 days of hospitalization.

Pre-existing cardiovascular diseases are associated to a worse prognosis in patients with SARS-CoV-2 infection (6). In our series, eight patients had previous cardiac conditions, but the autopsy did not reveal other lesions than those related with the underlying disease. No differences were observed in the evolution of these cases in comparison to patients without previous cardiac conditions but presenting other comorbidity.

In addition to coronary atherosclerosis, the most frequent cardiac pathological finding was left ventricular hypertrophy in 4 patients. While two of them had a previous diagnosis of hypertrophic cardiomyopathy, the other two patients were not previously diagnosed of any cardiac disease. We did not find other pathological findings in the heart of these 4 patients.

We found focal and slight lymphocytic inflammatory infiltrates in the epicardium of 3 patients. However, the mere presence of these aggregates is not indicative of active pericarditis. They were mainly placed in the subepicardial adipose tissue and they were not associated with vessels. For this reason, we cannot argue in favor of a systemic endothelialitis involving epicardial lymphatic micro-vessels. Because in our series 26 of patients (86.7%) were treated with mechanical ventilation and we only found chronic pericarditis in 3 of them (10%), our data do not support an association between both facts. Although 8 patients showed different degrees of pericardial effusion, we related this finding to the common hemodynamic alterations in terminal ICU patients. Pericardial effusion has been reported in up to 94% of patients dying from COVID-19 without evidence of pericarditis (12). Although clinical studies have reported some cases of pericarditis secondary to SARS-CoV-2 (14–17), most autopsy studies have not found severe acute pericarditis.

One of the most controversial issue in COVID-19 cardiac pathology is to know if myocarditis is a common manifestation of the disease (7, 11, 18–20). In our series, only one patient (3.3%) showed focal myocarditis characterized by both myocyte necrosis and inflammation in absence of ischemic changes. The frequency of the diagnosis of myocarditis varies among series, probably due to different diagnostic criteria among authors. According to cardiac autopsy guidelines of the European Association of Cardiovascular Pathology (AECVP) (21), focal presence of myocardial inflammatory infiltrates in the myocardic tissue in the absence of myocyte necrosis is not enough evidence for diagnosis of myocarditis. It also maintains that small fibrosis foci have no pathological significance.

Following the mentioned restrictive criteria, it seems that myocarditis is not a frequent manifestation of severe COVID-19. Halushka and Vander Heide (11) reviewed 22 articles about cardiovascular findings in autopsies samples from COVID-19 patients and they concluded that although inflammatory infiltrates were present in the myocardium of 7% of the 277 patients, only 1.7% had complete histopathological evidence of myocarditis. In the partially overlapping review by Roshdy et al. (10) including 316 patients, clear myocarditis meeting the Dallas criteria was described in only five cases, whereas 35 additional patients had focal inflammatory infiltrates. In their review, Kawakami et al. (12) specifically discussed the role of myocarditis in COVID-19 patients. The authors reviewed literature findings (some series also reviewed by Halushka and Vander Heide (11) and by Roshdy et al. (10) and found that myocarditis was an uncommon pathologic diagnosis occurring in 4.5% of highly selected cases undergoing autopsy or endomyocardial biopsy. In their own series of 16 autopsied patients, the authors observed myocardial inflammatory infiltrates in 31% of the patients, which were not associated with myocardial necrosis. The authors concluded that given the extremely low frequency of myocarditis and the unclear therapeutic implications, the use of endomyocardial biopsy to diagnose myocarditis in the setting of COVID-19 is not recommended. A recent series (22), where 5% of the patients developed new onset myocarditis, confirms these results.

In our study, inflammatory infiltrates within the myocardium associated to myocyte necrosis were identified in only one patient (3.3%). In our review of 277 reported autopsied hearts (not included in previously reported reviews and including our 30 patients), 20 (7.2%) showed evidence of myocarditis; however, the frequency was highly variable, ranging from 0 out of 97 patients in the series reported by Bryce et al. (9), to 9 out of 9 patients in the series reported by del Nonno et al. (23).

The role of SARS-CoV-2 as the direct cause of viral myocarditis remains to be established, similarly as has been observed in other organs, such as the brain, in which no direct viral brain damage has been proven in large autopsy case series (24). In our study, myocardial PCR was performed in order to detect SARS-CoV-2 in the myocardium of all autopsies, but only one case became positive. However, all cases showed at least one positive result in samples taken during the autopsy from the nasopharynx or lungs. In the patient with the positive RT-PCR in cardiac samples, no relevant cardiac histological alterations were identified, whereas in the patient with focal myocarditis, no virus was detected. Several studies have investigated the presence of SARS-CoV-2 in the myocardium using different techniques. In the review by Roshdy et al. (10), 105 hearts were studied for the presence of SARS-CoV-2 and 50 (47%) were positive. In our review, which included 60 patients in whom the presence of SARS-CoV-2 in the myocardium was investigated, 17 (28%) had a positive result. Differences among series can be explain by the time of disease evolution. Thus, the median of hospital stay was 5 and 6 days in Roshdy's and our review, respectively, but 29.5 days in our series. In fact, the only positive patient in our series had an illness duration of 9 days from symptoms onset to death. According to these data, myocarditis as an immunologically mediated phenomena rather than direct viral damage cannot be excluded. In this sense, immune-mediated myocarditis has been reported as a rare complication of COVID-19 mRNA vaccines (25).

One patient in this series had lesions of focal myocarditis due to Aspergillus fumigatus. Pulmonary aspergillosis can develop in severe COVID-19 patients. A review of 15 COVID-19-associated pulmonary aspergillosis (CAPA) clinical case series in the ICU reported 158 CAPA cases among 1,702 COVID-19 patients (9.3%, range between 0 and 33%). Only in four cases, CAPA was proven, while the majority had a probable or putative diagnosis (26). In a systematic review of autopsy series, the authors found 8 CAPA cases among 677 decedents (1.2%) (27). Cardiac lesions occurring in the setting of disseminated aspergillosis, as occurred in our patient, seem to be very unusual. Hanley et al. (14) reported a patient with an acute fungal pericarditis without characterization of the fungus.

Regarding thrombotic phenomena, SARS-CoV-2 infection has been associated with an increased thrombotic risk (28–30) and the presence of both micro and macrothrombi. We identified a patient with an atrial thrombus, but no cases of microthrombosis were observed. However, macro and microthrombosis were frequent in the lungs, even though all but three patients were being treated with prophylactic anticoagulation treatment. Thrombosis in COVID-19 patients has been related to the expression of ACE2 in endothelial tissue, which binds with SARS-CoV-2 causing direct endothelial damage and favoring thrombotic phenomena. The presence of thrombi has also been associated with an exaggerated immune response that triggers endothelial dysfunction and dysregulation. The frequency of both micro and macrothrombi varies largely among series (Supplementary Table 1). Thus, Pellegrini et al. (30) reported that 35% of patients in their series had myocardial necrosis and the most common cause associated with necrosis was the presence of microthrombi in 64% of cases. Bois et al. (31) also reported the presence of small vessel thrombosis in 80% of their patients. In contrast, other studies, including the series reported by Bryce et al. (9), who studied 97 patients, did not find heart vascular thrombosis. Since cardiac vascular pathology seems to be more frequent during the initial stage of the diseases (32), differences among series could be related, at least in part, with the time of evolution of the infection.

Regarding other histological findings, one of our elderly patients showed the presence of amyloid deposit within the myocardium that was positive for transthyretin. Other studies have identified the presence of cardiac amyloidosis in patients with COVID-19 (14, 33). Although not all series performed immunohistochemical studies, most cases, as the patient here reported and those reported by Menter et al. (34), are probably examples of senile amyloidosis. The frequency of cardiac amyloidosis is highly variable among series, ranging from 0 to 26.7%. In the reviews by Halushka and Vander Heide (11) and by Roshdy et al. (10) the frequency was 4 and 3.5%, respectively. In our own review, the frequency was 7.2%. Probably, differences among series were partially explained by the age of the patients included, since in our review the frequency of amyloidosis was high (14 to 26.7%) in the series in which the median age was 74 years or older but was low (0 to 7.3%) in those series with a median age lower than 70 years.

The limitations of our study include a relative low number of patients, who probably do not represent the complete spectrum of COVID-19 causes of dead. The use of two methods to study the hearts (partial and complete examination) is another limitation. However, we have not found differences between the pathological findings between both, except the presence of chronic epicardial inflammation, which has been more prevalent following the complete examination protocol. Finally, the lack of a control group of non-COVID-19 patients of similar age precludes any conclusion regarding if some lesions, such as collagen deposition, are increased in COVID-19 hearts.

Our series indicates that cardiac pathology is only modest in most patients and mainly consists of focal epicardial and myocardial inflammation, with little contribution of a direct effect of SARS-CoV-2. However, the frequency of these and other manifestations is highly variable among series suggesting that, in addition to biological variables, such as the time of evolution and methodological variables, like the extent of sampling, are responsible of these differences.

## Data Availability Statement

The original contributions presented in the study are included in the article/Supplementary Material, further inquiries can be directed to the corresponding author/s.

## Ethics Statement

The studies involving human participants were reviewed and approved by the Research Ethics Committee, Hospital Universitario Ramón y Cajal, Madrid, Spain (reference: Necropsias_Covid19; 355_20). Written informed consent for participation was not required for this study in accordance with the national legislation and the institutional requirements.

## Author Contributions

AF-G, HP-A, and JP contributed to conception and design of the study. AF-G, BP-M, and IR-C organized the database. IC-B, AN-C, DP, and JP wrote the first draft of the manuscript. RP, JZ, and JG wrote sections of the manuscript. All authors contributed to manuscript revision, read, and approved the submitted version.

## Funding

This work was supported by Instituto de Salud Carlos III (ISCIII) grant PI 19/01331, CIBERONC (grant CB16/12/00316), CIBERESP (grant CB06/02/0053), by the European Development Regional Fund. A way to achieve Europe (FEDER) and by MISP through the Investigator Initiated Studies Program (grant 2021/0224) and the Ramón y Cajal Institute for Health Research (IRYCIS) (COVID-19 Grant, 2020/0290).

## Conflict of Interest

The authors declare that the research was conducted in the absence of any commercial or financial relationships that could be construed as a potential conflict of interest.

## Publisher's Note

All claims expressed in this article are solely those of the authors and do not necessarily represent those of their affiliated organizations, or those of the publisher, the editors and the reviewers. Any product that may be evaluated in this article, or claim that may be made by its manufacturer, is not guaranteed or endorsed by the publisher.
