# Supplementary material for: Late Cardiac Pathology in Severe Covid-19. A Postmortem Series of 30 Patients
Source: Front Cardiovasc Med. 2021 Oct 15;8:748396. doi: 10.3389/fcvm.2021.748396 (PMC8555828; doi:10.3389/fcvm.2021.748396)
Supplement: Supplementary file 1 [file Data_Sheet_1.PDF]

Supplementary Material

Supplemental Table 1: Clinical and pathological findings of the series.

	**Halushka and Vander Heid** (1)	**Bryce et al.** (2)	**Bearse et al.** (3)	**Pellegrini et al.** (4)	**Haslbauer et al.** (5)	**Kawakami et al.** (6)	**Bois et al.** (7)	**Del Nonno et al.** (8)	**Jum'ha et al.** (9)	**Our series**
Autopsies	293	100	41	40	23	16	15	9	6	30
Autopsied hearts	277	97	41	40	23	16	15	9	6	30
<u>Demography</u>										
Male, n (%)	172 (62.1)	57 (57)	27 (65.9)	29 (72.5)	18 (78)	11 (68.7)	12 (80)	7 (77.7)	3 (50)	24 (80)
Age	75 (22-97) median (range)	66.1 (29.0-94.0) mean (range)	67 (21->89) median (range)	74 (65-81) median (IQR)	76 mean (SD)	70 (57-80) median (IQR)	78 (71-86) median (IQR)	69.9 (35-93) mean (range)	62 (53-73) mean (range)	69 (59-73.7) median (IQR)

	**Halushka and Vander Heid** (1)	**Bryce et al.** (2)	**Bearse et al.** (3)	**Pellegrini et al.** (4)	**Haslbauer et al.** (5)	**Kawakami et al.** (6)	**Bois et al.** (7)	**Del Nonno et al.** (8)	**Jum'ha et al.** (9)	**Our series**
BMI	26.9 (14.9-59) median (range)	NE	(20-70) (range)	27.7 (24.7-29.3) median (IQR)	27 (26-35) median (IQR)	NE	26.15 (19.5-41) mean (range)	NE	32.96 (25.2-38.4) mean (range)	26.8 (25.2-30.8) [n=16] median (IQR)
Admission length	10 (1-51) median (range)	14.0 (0.0-87.0) mean (range)	13 (1-39) median (range)	NE	7 (5-12) median (IQR)	6 (3-9.5) median (IQR)	NE	19.5 (10-31) mean (range)	NE	29.5 (18-36) median (IQR)
CV disease, n (%)	153 (55.2)	13 (13)	2 (4.9)	12 (30)	14 (61)	4 (25)	NE	2 (22.2)	3 (50)	6 (20)
Hypertension, n (%)	152 (54.9)	62 (62)	27 (65.9)	29 (72.5)	21 (91)	NE	NE	0	4 (66.6)	13 (43.3)
Diabetes, n (%)	89 (32.1)	43 (43)	14 (34.1)	11 (27.5)	11 (48)	NE	NE	NE	4 (66.6)	3 (10)
Obesity, n (%)	44 (15.9)	11 (11)	NE	NE	NE	NE	3 (20)	NE	5 (83.3)	7 (23.3)
COPD, n (%)	70 (25.3)	5 (5)	NE	3 (7.5)	4 (17)	NE	NE	1 (11.1)	3 (50)	1 (3.3)

	**Halushka and Vander Heid** (1)	**Bryce et al.** (2)	**Bearse et al.** (3)	**Pellegrini et al.** (4)	**Haslbauer et al.** (5)	**Kawakami et al.** (6)	**Bois et al.** (7)	**Del Nonno et al.** (8)	**Jum'ha et al.** (9)	**Our series**
<u>Cardiovascular findings</u>										
(+) RT-PCR in myocardium, n (%)	NE	NE	NE	NE	14 (60)	2 (12.5)	0	NE	NE	1 (3.3)
Heart weight	483 (250-1070) median (range)	NE	(204-738) (range)	NE	468 (430-550) median (range)	404 (368-470) median (IQR)	443.1 (286.3-545) median (range)	NE	455 (300-640) mean (range)	450 (399-531) [n=13] median (IQR)
Amyloidosis, n (%)	11 (4)	0	3 (7.3)	6 (14.3)	6 (26)	0	4 (26.7)	0	0	1 (3.3)
Myocardial inflammation/Myocarditis, n (%)	20 (7.2)	0	4 (9.8)	0	1 (4)	0	5 (33.3)	9 (100)	0	1 (3.3)
Epicardial inflammation/Pericarditis, n (%)	19 (6.9)	31 (32)	9 (22)	0	0	10 (62.5)	NE	8 (88.8)	1 (16.6)	4 (13.3)

	**Halushka and Vander Heid** (1)	**Bryce et al.** (2)	**Bearse et al.** (3)	**Pellegrini et al.** (4)	**Haslbauer et al.** (5)	**Kawakami et al.** (6)	**Bois et al.** (7)	**Del Nonno et al.** (8)	**Jum'ha et al.** (9)	**Our series**
Small vessel thrombi, n (%)	30 (10.8)	3 (3.1)	5 (12.2)	9 (22.5)	0	0	12 (80)	0	0	0
Macrothrombi, n (%)	53 (19.1)	0	NE	3 (7.5)	0	0	2 (13.3)	0	0	1 (3.3)
Acute myocardial infarction, n (%)	13 (4.7)	0	NE	3 (7.5)	1 (4)	3 (19)	2 (13.3)	0	0	0

1 References

1. Halushka MK, Vander Heide RS. Myocarditis is rare in COVID-19 autopsies: cardiovascular findings across 277 postmortem examinations. Cardiovasc Pathol. 2021;50:107300.

2. Bryce C, Grimes Z, Pujadas E, Ahuja S, Beasley MB, Albrecht R, et al. Pathophysiology of SARS-CoV-2: the Mount Sinai COVID-19 autopsy experience. Mod Pathol. 2021 Apr 1;1–12.

3. Bearse M, Hung YP, Krauson AJ, Bonanno L, Boyraz B, Harris CK, et al. Factors associated with myocardial SARS-CoV-2 infection, myocarditis, and cardiac inflammation in patients with COVID-19. Mod Pathol. 2021 Jul;34(7):1345–57.

4. Pellegrini D, Kawakami R, Guagliumi G, Sakamoto A, Kawai K, Gianatti A, et al. Microthrombi as a Major Cause of Cardiac Injury in COVID-19: A Pathologic Study. Circulation. 2021 Mar 9;143(10):1031–42.

5. Haslbauer JD, Tzankov A, Mertz KD, Schwab N, Nienhold R, Twerenbold R, et al. Characterisation of cardiac pathology in 23 autopsies of lethal COVID-19. J Pathol Clin Res. 2021 Jul;7(4):326–37.

6. Kawakami R, Sakamoto A, Kawai K, Gianatti A, Pellegrini D, Nasr A, et al. Pathological Evidence for SARS-CoV-2 as a Cause of Myocarditis: JACC Review Topic of the Week. J Am Coll Cardiol. 2021 Jan 26;77(3):314–25.

7. Bois MC, Boire NA, Layman AJ, Aubry M-C, Alexander MP, Roden AC, et al. COVID-19-Associated Nonocclusive Fibrin Microthrombi in the Heart. Circulation. 2021 Jan 19;143(3):230–43.

8. del Nonno F, Frustaci A, Verardo R, Chimenti C, Nicastri E, Antinori A, et al. Virus-Negative Myopericarditis in Human Coronavirus Infection. Circ Heart Fail. 2020 Nov 1;13(11):e007636.

9. Jum’ah H, Loeffler A, Tomashefski JF. Histopathological findings in the hearts of COVID-19 autopsies: a letter to Cardiovascular pathology journal editor in response to Halushka et al. 2020. Cardiovasc Pathol. 2021 May;52:107333.
